# Vanilla LSTM Predictive Maintenance Model for Scientific Research Facilities

**DOI:** 10.3390/s26144581

**Published:** 2026-07-20

**Authors:** Edward Nkadimeng, Mpho Gololo, Manal Karmoude, Nieldane Stodart, Mukesh Kumar, Bruce Mellado

**Affiliations:** 1 School of Physics and Institute for Collider Particle Physics, University of the Witwatersrand, Johannesburg 2050, South Africa; manal.karmoude@cern.ch (M.K.); bruce.mellado@wits.ac.za (B.M.); 2 Department of Electrical and Electronic Engineering Science, Faculty of Engineering and the Built Environment, University of Johannesburg, Auckland Park, Johannesburg 2006, South Africa; mphog@uj.ac.za; 3 iThemba LABS, National Research Foundation, Old Faure Road, Somerset West 7129, South Africa; nieldane@tlabs.ac.za

**Keywords:** predictive maintenance, long short-term memory, LSTM, failure prediction, multivariate sensor data, scientific equipment reliability, Industry 4.0, anomaly detection, failure score, iThemba LABS

## Abstract

Ensuring the reliability and operational efficiency of critical scientific equipment is a central challenge in high-stakes research environments such as nuclear physics laboratories and particle accelerator facilities. Unexpected failures entail significant financial cost and prolonged interruptions to experimental programmes. We present a predictive maintenance (PdM) framework built around a two-layer Vanilla Long Short-Term Memory (LSTM) network trained on multivariate sensor streams collected at NRF-iThemba LABS between January 2021 and December 2023. Four channels, namely supply voltage, vibration velocity, differential pressure, and rotational speed, were recorded at 5 min intervals using a suite of industrial-grade transducers (power quality analyser, IEPE accelerometers, differential pressure transmitters, and proximity encoders) feeding a multi-channel data-acquisition chassis via OPC-UA, yielding a time-synchronised dataset of 315,360 observations. A normalised failure score converts the binary classifier output into a continuous, interpretable health indicator that supports tiered scheduling of maintenance. The Vanilla LSTM achieved a test-set F1-score of 75% and an area under the receiver-operating-characteristic curve (AUC) of 0.856, outperforming five competing architectures (PCA/$T^{2}$, Random Forest, Deep Neural Network, LSTM Autoencoder, and Bidirectional LSTM Autoencoder), and delivered a mean failure lead time of (42.3±7.2) h, exceeding the 36 h engineering requirement for proactive maintenance scheduling.

## 1. Introduction

Scientific research facilities including nuclear physics laboratories, isotope production plants, and particle accelerator complexes depend on uninterrupted operation of precision electromechanical equipment. At NRF-iThemba LABS in South Africa, the separated-sector cyclotron and wide-band separator operate drive motors, vacuum pumps, power supplies, and beam-line instrumentation under demanding duty cycles [1]. A single unplanned failure can delay beam-time allocations by weeks and impose repair costs that exceed scheduled maintenance by an order of magnitude [2,3]. Reactive strategies incur unplanned downtime and emergency repair premiums, while time-based preventive strategies replace components before failure, wasting residual service life and interrupting experimental programmes [4,5].

Predictive maintenance (PdM) bases intervention decisions on the real-time health state of each asset, inferred from continuous sensor monitoring and machine-learning models that detect incipient degradation before it becomes critical [6]. At iThemba LABS, the deployment of OPC-UA-compatible transducers and a multi-channel data-acquisition chassis [7] made continuous multivariate monitoring at 5 min resolution operationally viable from 2021, generating rich, time-synchronised sensor streams across four physical channels. The condition-monitoring system targets the dominant failure modes of this equipment class: bearing wear and pitting, rotational imbalance and shaft misalignment, electrical supply degradation in the motor-drive power supplies, and loss of cooling or vacuum performance. These modes manifest as coupled, gradually developing signatures across the vibration, voltage, pressure, and rotational-speed channels, which is the basis for the multivariate approach detailed in Section 3.1.

Classical anomaly-detection methods based on Principal Component Analysis (PCA) with Hotelling’s $T^{2}$ statistic discard temporal ordering and assume linear Gaussian sensor relationships assumptions violated by the non-stationary dynamics of degrading electromechanical systems [8]. Ensemble methods such as Random Forest (RF) capture non-linear interactions but require hand-crafted temporal features that do not generalise automatically across operating regimes [9]. Long Short-Term Memory (LSTM) networks [10], designed to learn long-range temporal dependencies through a gated cell-state mechanism, are well suited for PdM on multivariate sensor streams. While LSTM Autoencoders and Bidirectional (BiLSTM) variants have attracted interest [11,12], the Vanilla LSTM operating in direct classification mode is simpler, has lower latency, and is deployable without GPU hardware, yet it has been under-evaluated in scientific-equipment settings.

The purpose of this study is to determine whether a deliberately simple, deployable Vanilla LSTM operating in direct classification mode can provide sufficient predictive lead time for proactive maintenance of scientific research equipment and to benchmark it rigorously against both classical and more complex deep-learning alternatives on real facility data. To this end, this paper makes four contributions:

(i) A fully specified industrial sensor acquisition pipeline for NRF-iThemba LABS equipment;
(ii) A complete PdM framework from raw sensor ingestion to deployment-ready health scoring;
(iii) A normalised failure score metric supporting tiered maintenance decision-making;
(iv) A systematic six-model comparison demonstrating that the Vanilla LSTM achieves the highest predictive accuracy, AUC, and failure lead time.

The remainder of this paper is organised as follows. Section 2 reviews statistical, machine-learning, and deep-learning approaches to equipment health monitoring and identifies the gap addressed here. Section 3.1 describes the instrumented equipment, transducer suite, and data-acquisition architecture. Section 4 details data preprocessing, feature engineering, the six model architectures, and the training protocol. Section 5 reports predictive accuracy, ROC and confusion-matrix analyses, failure-score validation, and lead-time results, including feature-ablation and noise-robustness studies. Section 6 interprets these findings and states the limitations, and Section 7 concludes this paper.

## 2. Literature Review

### 2.1. Statistical Process Monitoring

PCA statistical process monitoring, originally formalised by Hotelling [13] and later extended to multivariate batch processes by Nomikos and MacGregor [8], remains one of the most widely deployed baseline approaches in industrial condition monitoring. Its appeal lies in its simplicity and interpretability, particularly in environments where process variables are strongly correlated. However, several limitations are well established. The assumption of linearity restricts its ability to capture complex system dynamics, while its formulation is inherently static and therefore insensitive to temporal degradation patterns. In addition, performance is often highly sensitive to the choice and number of retained principal components. These limitations have motivated a shift towards data-driven and learning-based approaches [3,14].

### 2.2. Machine Learning for Equipment Health Monitoring

Machine learning methods have been widely explored as a means of addressing these shortcomings. For example, Random Forest models have demonstrated high-precision disk failure prediction in large-scale data centre environments [9]. However, such approaches typically rely on carefully engineered temporal windows, which do not transfer easily across different equipment classes or operating regimes. Similarly, Support Vector Machines have shown strong performance in controlled settings, but their computational cost scales poorly with high-dimensional time-series inputs, limiting their applicability in real-time monitoring contexts [14]. In practice, these methods often require significant feature engineering and domain-specific tuning, which constrains their generalisability. Broader surveys of data-driven predictive maintenance reach the same conclusion, noting a persistent reliance on hand-crafted features and limited public benchmark data across industrial domains [15].

### 2.3. Deep Learning for Predictive Maintenance

Deep learning approaches have increasingly been adopted to overcome these limitations, particularly in settings where large volumes of labelled data are available. A comprehensive benchmark across 97 time-series classification datasets demonstrated that recurrent architectures, including LSTMs, consistently outperform classical methods under such conditions [11]. In the context of machine health monitoring, it has been shown that deep learning models operating directly on raw vibration signals can match or exceed traditional feature-engineering-based approaches [12]. More recent comparative studies confirm this trend: a systematic evaluation across three industrial datasets reported that hybrid CNN–LSTM architectures achieve the strongest fault-prediction performance and used ablation studies to identify the feature families most influential to accuracy [16]. Hybrid recurrent designs combining convolutional feature extraction with bidirectional LSTM layers have likewise improved remaining useful life estimation on multi-sensor data [17], and attention-based transformer models have since been proposed as an alternative for capturing long-range dependencies in degradation signals [18,19]. Furthermore, LSTM-based models have achieved improved accuracy in remaining useful life estimation compared to support vector regression on benchmark datasets such as NASA C-MAPSS [20]. Unsupervised approaches, such as LSTM autoencoders, have also been proposed for anomaly detection, including recent hybrid frameworks that pair an LSTM autoencoder for anomaly screening with a supervised classifier for fault prediction under realistic class imbalance [21]. However, these reconstruction-based methods often conflate novelty with failure, leading to elevated false-alarm rates in systems where the normal operating envelope evolves over time [22]. In this work, a Vanilla LSTM is instead deployed in a direct classification framework. This formulation avoids the ambiguity inherent in reconstruction- based methods and, as demonstrated in the subsequent sections, provides improved predictive performance while maintaining operational interpretability.

In summary, the literature reveals a consistent trade-off. Statistical process monitoring is interpretable but discards temporal structure and assumes linear Gaussian behaviour; classical machine-learning methods capture non-linear relationships but depend on hand-crafted features that generalise poorly across operating regimes; and deep-learning methods learn temporal structure automatically but are most commonly deployed either as remaining-useful-life regressors requiring run-to-failure trajectories or as reconstruction-based anomaly detectors prone to false alarms under evolving operating envelopes. What is missing is a systematic demonstration, on real scientific-facility data with realistic class imbalance, that a standard recurrent classifier can deliver actionable failure lead time within a deployable pipeline, benchmarked against both classical and deep-learning alternatives. The present work addresses this gap directly.

## 3. Sensor Hardware and Data Acquisition

### 3.1. Instrumented Equipment

**Figure 1:**
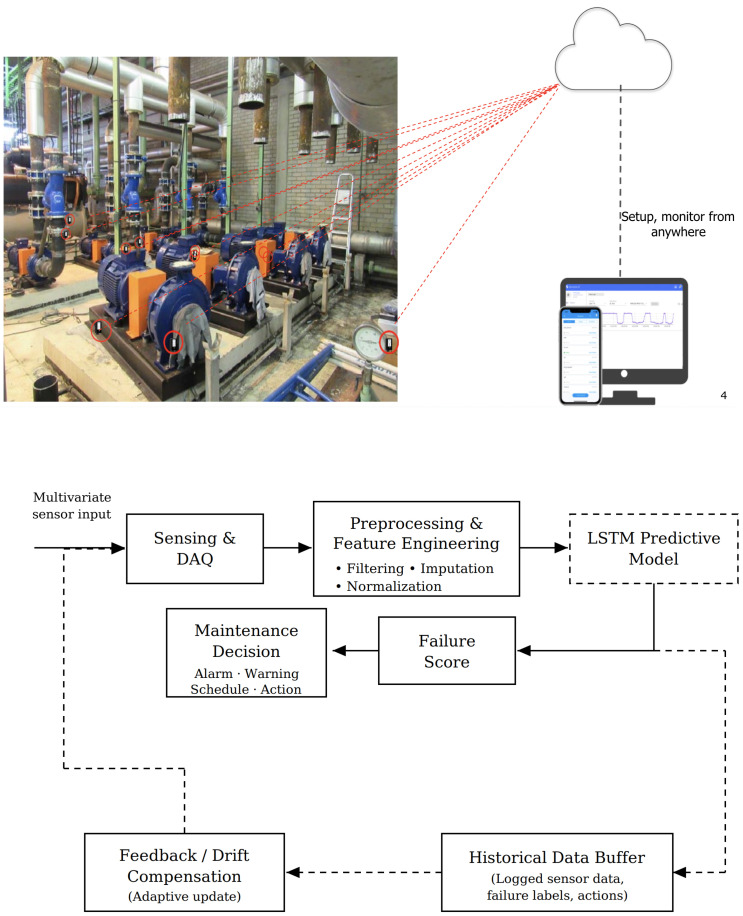
Sensor deployment and predictive maintenance architecture for the NRF-iThemba LABS system. (**Top**): physical deployment, showing transducers installed on the critical pump and vacuum equipment. (**Bottom**): data-flow schematic. Multivariate sensor input from the four transducer types (power quality analyser, accelerometer, differential pressure transmitter, and proximity encoder) measuring supply voltage, vibration velocity, differential pressure, and rotational speed, respectively, enters the sensing and data-acquisition (DAQ) stage at 5 min cadence. Solid boxes denote the forward processing path and the dashed box denotes the trained LSTM predictive model; solid arrows indicate the forward data flow and dashed arrows indicate the feedback and adaptive-retraining loop. Acquired streams pass through preprocessing and feature engineering (filtering, imputation, and normalisation) to the LSTM predictive model, which publishes a continuous failure score that is converted into a tiered maintenance decision. Logged sensor data, failure labels, and recorded actions accumulate in a historical data buffer, maintained within the facility Supervisory Control and Data Acquisition (SCADA) infrastructure [24], that supports feedback and drift compensation of the deployed model.

This study focuses primarily on water cooling pump systems, vacuum subsystems, and associated power supplies within the NRF-iThemba LABS cyclotron facility. These systems form the operational backbone of the accelerator, where stable thermal management, vacuum integrity, and electrical reliability are tightly coupled to beam performance. Although functionally different, these assets exhibit a common set of degradation mechanisms under continuous operation. In water pumps, bearing wear, cavitation, and seal degradation dominate, while vacuum systems are more susceptible to pressure instability, leakage, and pump inefficiencies. Power supplies, particularly those driving motors and auxiliary systems, are prone to insulation breakdown, harmonic distortion, and electrical imbalance. A key observation is that these failure modes do not evolve in isolation. Mechanical degradation in pumps, for example, often manifests as both vibration anomalies and pressure fluctuations, while electrical faults propagate through power quality signatures. This motivates a monitoring strategy in which multiple sensing channels are jointly analysed to capture the coupled nature of system degradation, as seen in Figure 1.

The monitored fleet comprises multiple nominally identical units of each asset class (eight water-cooling pump sets, four vacuum subsystems, and their associated motor-drive power supplies). Although units within a class share a design and are therefore expected to follow a common reliability distribution, they operate under differing duty cycles and thermal loads depending on their position in the cooling and vacuum circuits. We therefore treat each asset class as a single type for labelling and feature construction, while pooling observations across units within a class to increase the number of observed degradation episodes. This pooling is appropriate given the shared failure mechanisms described above, but we note in Section 6 that unit-to-unit variability remains a source of residual heterogeneity.

### 3.2. Transducer Suite

#### 3.2.1. Voltage and Power Quality

Electrical behaviour is monitored using a Fluke 435-II three-phase power quality analyser (Fluke Corporation, Everett, WA, USA) installed at each motor control centre. The instrument provides true RMS voltage, power factor, and total harmonic distortion (THD) measurements at a sampling rate of 10 kHz, with an accuracy of ±(0.1%rdg+2cts), compliant with IEC 61010-1 CAT IV requirements. Beyond steady-state monitoring, the analyser captures transient phenomena including voltage sag, swell, and harmonic distortion. These effects are closely associated with early-stage electrical faults, such as winding insulation degradation and inter-turn short circuits in induction machines [23]. As such, power quality measurements form a primary diagnostic channel for incipient electrical failure.

#### 3.2.2. Vibration

Mechanical condition is assessed using PCB Piezotronics 622B01 IEPE accelerometers (PCB Piezotronics, Depew, NY, USA) mounted on bearing housings. These sensors provide a sensitivity of 100 mV/g (±5%) over a bandwidth of 0.5 Hz to 10,000 Hz, with a dynamic range of 0.001 g to 50 g.

Acceleration signals are digitally integrated to obtain vibration velocity (mm s^−1^) and band-pass filtered to the 10 Hz to 1000 Hz range, enabling direct comparison with ISO 10816-3 [25] severity classifications. The velocity root-mean-square in this band, $v_{\mathrm{rms}}\;\left[10-1000\;\mathrm{Hz}\right]$, is retained as the primary vibration indicator because it aligns with the facility’s existing ISO 10816-3 alarm thresholds and is robust to the 5 min acquisition cadence. We acknowledge that high-frequency acceleration envelopes in the 0.5 kHz to 10 kHz band are more sensitive to early bearing pitting; however, resolving these signatures requires continuous high-rate streaming that the present 5 min historian cadence does not support. The acceleration-derived velocity band therefore represents a deliberate trade-off favouring compatibility with the deployed SCADA infrastructure, and high-frequency envelope analysis is identified as a direction for future instrumentation upgrades. In this context, vibration serves as a sensitive indicator of mechanical degradation, including imbalance, misalignment, and bearing defects.

#### 3.2.3. Pressure

Process-level behaviour is monitored using Emerson Rosemount 3051C differential pressure transmitters (Emerson Electric, St. Louis, MO, USA) deployed on cooling-water and control-air circuits. The sensors operate over a 0–250 kPa range with a span accuracy of ±0.065%, providing a standard 4–20 mA output. Observed pressure deviations are indicative of system-level anomalies such as filter blockage, pump cavitation, or seal degradation, and therefore provide an indirect but critical measure of process integrity.

#### 3.2.4. Rotational Speed

Rotational dynamics are captured using Pepperl + Fuchs KFD2 proximity encoders (Pepperl + Fuchs, Mannheim, Germany) coupled to 60-tooth target wheels. Pulse trains are converted to rotational speed (RPM) with an accuracy of ±0.1%. These measurements enable the detection of load fluctuations, drive instability, and emerging mechanical faults.

### 3.3. Signal Conditioning and DAQ

All analogue sensor outputs are interfaced through a National Instruments cDAQ-9178 chassis (National Instruments, Austin, TX, USA) equipped with NI 9205 (16-bit ADC) modules for analogue acquisition, NI 9411 digital inputs for encoder signals, and NI 9485 relay outputs for alert actuation. Signal integrity is maintained through galvanic isolation and 1 kHz anti-alias filtering prior to digitisation. The system is designed to ensure consistent, high-fidelity acquisition across all channels under operational conditions.

Data are streamed via OPC-UA(IEC 62541) [7] to the facility SCADA historian, where they are synchronised using network time protocol (NTP) with an accuracy of ±1 ms. This provides a unified temporal framework for subsequent analysis and model deployment.

### 3.4. Dataset Overview

The dataset spans January 2021 to December 2023 at a fixed sampling interval of 5 min, yielding 315,360 time-aligned observations per channel. In addition to continuous sensor measurements, event-driven failure logs and SCADA error codes are recorded asynchronously and subsequently aligned to the nearest preceding timestamp.

This combination of regularly sampled process variables and irregular failure annotations reflects the realities of industrial operation, where measurements are continuous but failure reporting is inherently discrete and operator driven. As a result, the dataset captures not only steady-state behaviour but also transient and pre-failure dynamics across multiple timescales. Table 1 summarises the recorded variables. The four primary channels voltage, vibration, pressure, and rotational speed represent complementary views of system behaviour, spanning electrical, mechanical, and process domains. Importantly, failure events are relatively sparse, leading to a naturally imbalanced dataset characteristic of predictive maintenance problems.

## 4. Materials and Methods

### 4.1. Data Preprocessing

#### 4.1.1. Timestamp Alignment and Label Propagation

**Figure 2:**
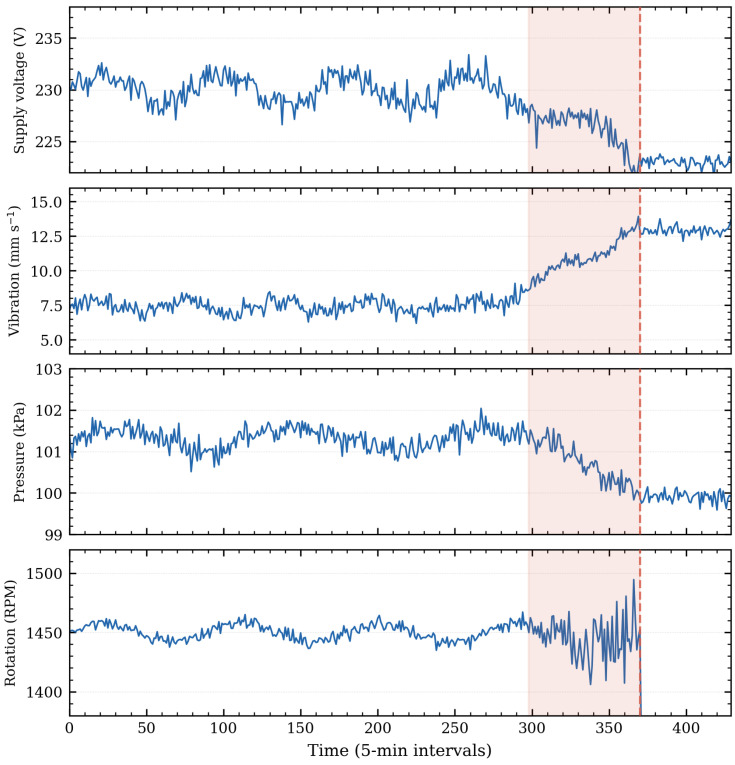
Representative multivariate sensor trajectories prior to a recorded failure event (dashed red line). The shaded region denotes the 6 h label-propagation window (Equation (1)). From top: supply voltage (Fluke 435-II), vibration (PCB 622B01), pressure (Rosemount 3051C), and rotation (P + F KFD2).

A key challenge in this dataset is the mismatch between continuous sensor sampling and discrete failure logging. Failure events are recorded at the time of detection or intervention, rather than at the true onset of degradation as seen in Figure 2. To address this, each failure event is mapped to the nearest preceding observation, and a forward-looking label propagation strategy is adopted. Specifically, a rolling window of $W_{\mathrm{lead}}=72$ steps (6 h) is used to label all observations preceding a failure as belonging to the pre-failure class:

(1) $$y_{t}=\left\{\begin{array}{cc} 1 & \mathrm{if}\;\exists \;k\in \left[t,\;t+W_{\mathrm{lead}}\right]\;s.t.\;f_{k}=1,\\ 0 & \mathrm{otherwise}. \end{array}\right.$$

This formulation reflects the practical objective of predictive maintenance: not merely detecting failure, but identifying actionable precursor states with sufficient lead time for intervention. The choice of a 6 h window is informed by operational constraints, including typical response times for maintenance scheduling and system shutdown procedures. It is important to distinguish between the training label definition and the reported failure lead time. While the former defines the supervision signal, the latter introduced in Section 5.5 quantifies the actual time between the first threshold crossing and the recorded failure event.

#### 4.1.2. Missing Data and Noise Handling

Industrial sensor streams are subject to intermittent data loss and measurement noise arising from communication latency, sensor drift, and electromagnetic interference. To maintain temporal continuity, short gaps in continuous channels are filled using linear interpolation, while categorical error logs are forward-filled to preserve event persistence.

Observations with more than 10% missing features are discarded to avoid introducing bias through excessive imputation. Residual high-frequency noise is attenuated using a moving-average filter with a window size of w=3:

(2) $${\tilde{X}}_{t}=\frac{1}{w}\sum_{i=0}^{w-1}X_{t-i}.$$

This level of smoothing was found to suppress spurious fluctuations while preserving the slower dynamics associated with degradation processes, which typically evolve over timescales of tens of minutes to hours.

#### 4.1.3. Feature Engineering

Feature engineering is designed to capture both instantaneous behaviour and temporal evolution. In addition to raw sensor values, rolling statistics (mean and standard deviation) are computed over 1, 3, and 6 h windows to encode short- and medium-term trends.

First-order differences $\Delta X_{t}$ capture local dynamics and abrupt changes, while short-time Fourier magnitudes of the vibration signal provide a compact representation of frequency-domain behaviour associated with mechanical faults. These frequency-domain features are computed from the same 10 Hz to 1000 Hz band-limited velocity signal used for the $v_{\mathrm{rms}}$ indicator; the $v_{\mathrm{rms}}$ scalar summarises overall severity, whereas the short-time Fourier magnitudes preserve the spectral distribution within that band. Error codes are one-hot encoded and augmented with rolling frequency counts, allowing the model to incorporate operational context alongside physical measurements.

This combination of time-domain, frequency-domain, and event-based features is intended to reflect the multi-scale nature of degradation, where early signatures may be subtle and distributed across multiple channels.

#### 4.1.4. Dimensionality Analysis

**Figure 3:**
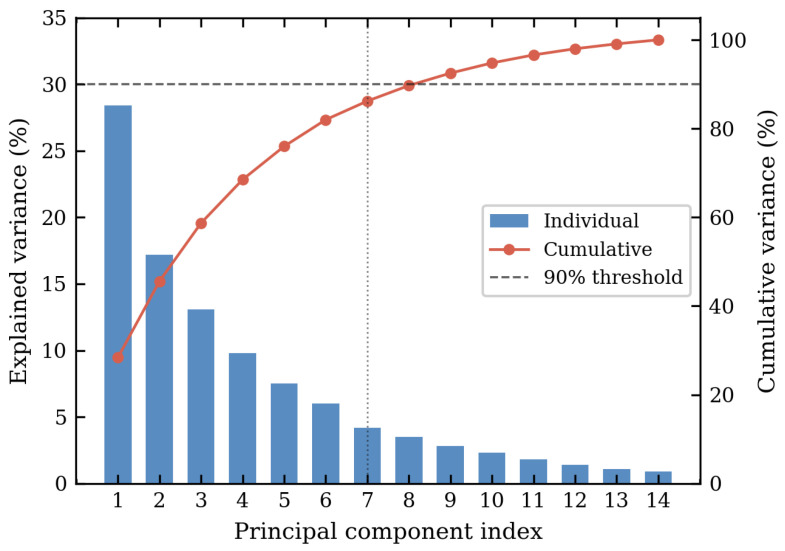
Individual explained variance (bars, left axis) and cumulative explained variance (line, right axis) versus principal component index. The dashed line marks the 90% threshold, first exceeded at eight components; the vertical dotted line indicates the corresponding number of components retained.

Principal Component Analysis (PCA) is applied to the engineered feature matrix to assess intrinsic dimensionality. Eight principal components are sufficient to retain 90% of the total variance (Figure 3), indicating that the data lie on a low-dimensional manifold despite the high-dimensional feature space.

However, explicit projection onto this subspace prior to LSTM training does not yield performance gains. This suggests that the LSTM is effectively learning an internal representation of the underlying structure, consistent with its ability to perform implicit feature selection and temporal integration.

#### 4.1.5. Exploratory Correlation Analysis

**Figure 4:**
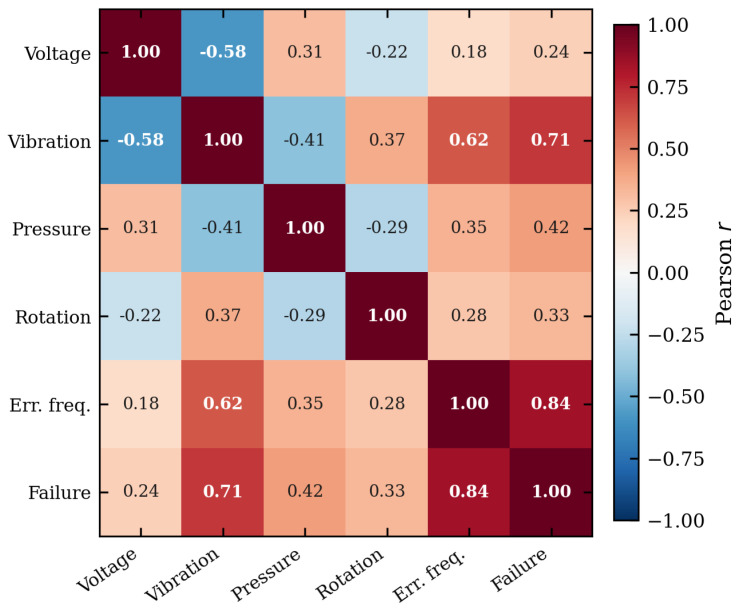
Pearson correlation matrix for sensor channels, error code frequency, and the binary failure label. Values exceeding |r|=0.55 are annotated in white.

The Pearson correlation matrix (Figure 4) highlights strong interdependencies between sensing modalities. In particular, vibration exhibits a pronounced negative correlation with voltage (r=−0.58), suggesting coupled electrical and mechanical degradation processes.

Furthermore, vibration and error code frequency show the strongest association with the failure label (r=0.71 and r=0.84), reinforcing their role as primary indicators of system health. These observations are consistent with expected failure behaviour in rotating machinery, where mechanical imbalance and electrical anomalies often co-evolve.

### 4.2. Model Architectures

Six architectures are evaluated on identical data partitions.

#### 4.2.1. PCA with Hotelling’s $T^{2}$ (Baseline)

The PCA baseline projects each standardised feature vector onto its principal components and monitors the Hotelling’s $T^{2}$ statistic, defined as

(3) $$T^{2}=z^{⊤}\Lambda^{-1}z,$$

where z is the PCA score vector, and Λ is the diagonal eigenvalue matrix. An observation is flagged when $T^{2}$ exceeds the *F*-distribution critical value at the 5% significance level.

#### 4.2.2. Random Forest

The Random Forest baseline uses 200 trees with a 72-step lag-feature window, a maximum depth of 15, a minimum of 2 samples per leaf, and class weights inversely proportional to class frequency.

#### 4.2.3. Deep Neural Network

Three fully connected layers (512–256–128 units), ReLU activations, batch normalisation, dropout p=0.3, and sigmoid output.

#### 4.2.4. Vanilla LSTM (Proposed)

A two-layer LSTM (128 units per layer) processes sequences of length L=72 steps (6 h receptive field). This length is selected from the feature autocorrelation analysis (Figure 2), in which the predictive autocorrelation of the engineered features decays to insignificance beyond approximately six hours. Sequence lengths larger than L=72 were found to add input dimensionality and computational cost without supplying additional predictive signal, while also increasing the minimum data latency that must elapse before a prediction can be issued; the 6 h window therefore represents the most favourable trade-off between predictive information and operational responsiveness. Here, “Vanilla LSTM” denotes the standard formulation with input, forget, and output gates and no peephole connections, corresponding to the baseline architecture characterised in the systematic study of LSTM variants by Greff et al. [26]; we adopt this configuration in direct (many-to-one) classification mode rather than as an autoencoder. The cell dynamics follow the standard LSTM formulation [10]:

(4) $$\begin{array}{cc} i_{t} & =\sigma (W_{xi}x_{t}+W_{hi}h_{t-1}+b_{i}), \end{array}$$

(5) $$\begin{array}{cc} f_{t} & =\sigma (W_{xf}x_{t}+W_{hf}h_{t-1}+b_{f}), \end{array}$$

(6) $$\begin{array}{cc} o_{t} & =\sigma (W_{xo}x_{t}+W_{ho}h_{t-1}+b_{o}), \end{array}$$

(7) $$\begin{array}{cc} c_{t} & =f_{t}⊙c_{t-1}+i_{t}⊙\tanh\left(W_{xc}x_{t}+W_{hc}h_{t-1}+b_{c}\right), \end{array}$$

(8) $$\begin{array}{cc} h_{t} & =o_{t}⊙\tanh\left(c_{t}\right). \end{array}$$

**Figure 5:**
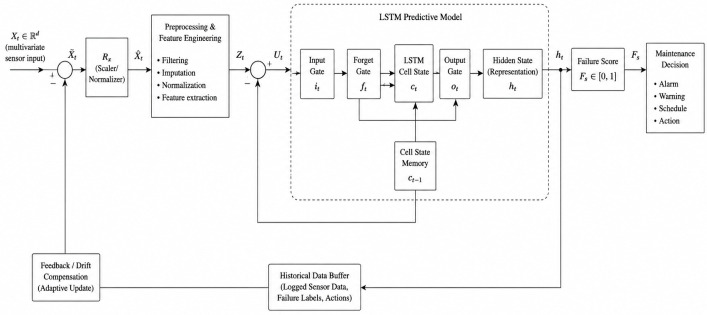
Vanilla LSTM predictive maintenance pipeline. Multivariate sensor inputs pass through preprocessing before entering the two-layer LSTM. The final hidden state is mapped to a normalised failure score $F_{s}\in \left[0,1\right]$ by a dense sigmoid layer.

Dropout p=0.2 is applied between layers. The final hidden state $h_{T}$ passes through a dense sigmoid layer to produce the class probability $p_{t}$. Figure 5 shows the full pipeline.

#### 4.2.5. LSTM Autoencoder and BiLSTM Autoencoder

The LSTM Autoencoder uses an encoder–decoder structure trained on normal sequences; reconstruction error serves as the anomaly score. The BiLSTM Autoencoder extends this by processing sequences in both temporal directions, doubling the effective receptive field.

### 4.3. Training Protocol

**Figure 6:**
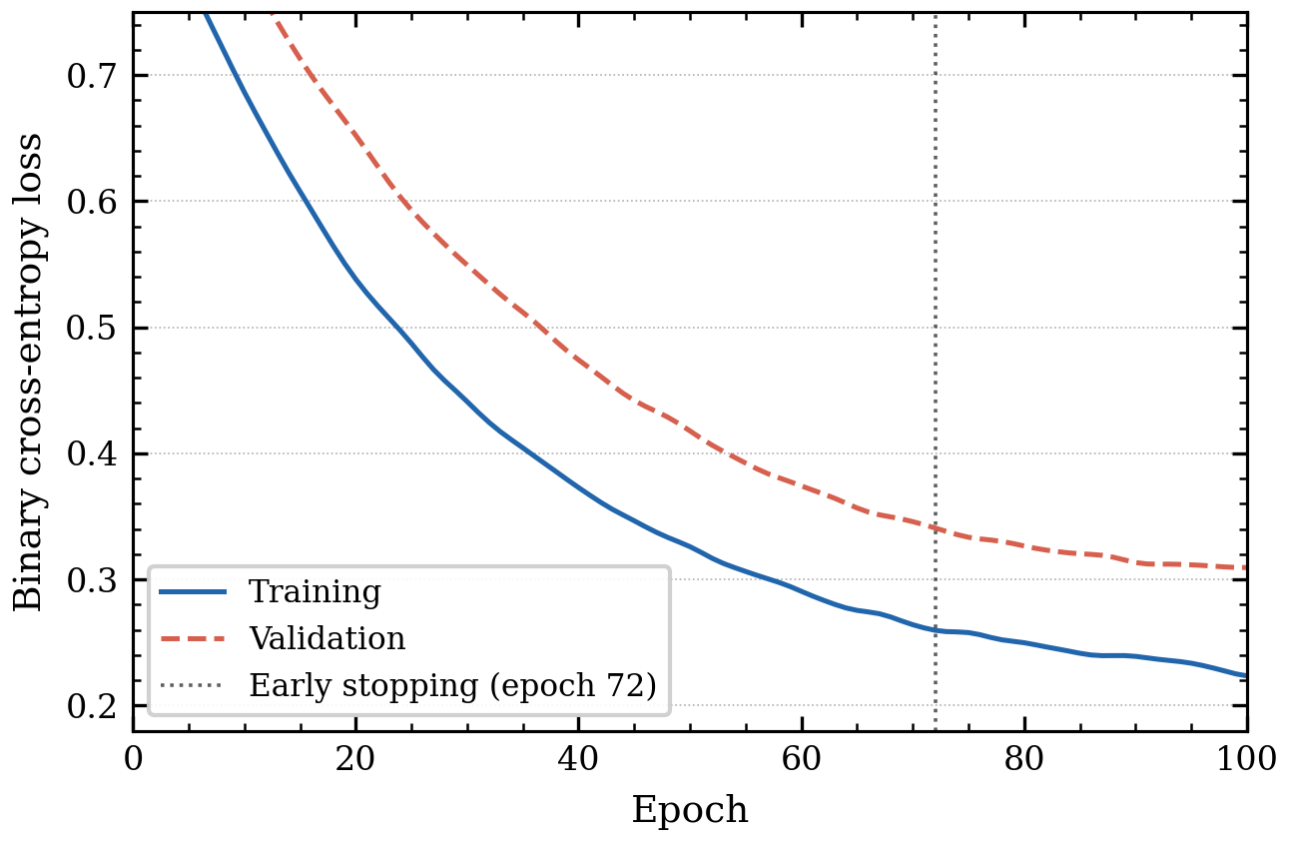
Binary cross-entropy loss during training (solid) and validation (dashed). The dotted vertical line marks the epoch selected by early stopping.

Data are partitioned 60:20:20 (train:validation:test) using stratified sampling to preserve the class imbalance ratio (≈12:1 normal:failure). Imbalance is addressed by class-weighted binary cross-entropy:

(9) $$L=-\frac{1}{N}\sum_{i=1}^{N}\left[w_{+}y_{i}\log{\hat{y}}_{i}+w_{-}\left(1-y_{i}\right)\log\left(1-{\hat{y}}_{i}\right)\right],$$

with $w_{+}$, $w_{-}$ inversely proportional to class frequency. All neural models use Adam ($\eta =10^{-3}$, $\beta_{1}=0.9$, $\beta_{2}=0.999$, batch size 64) with early stopping (patience 15 epochs). All models were implemented in Python 3.x using TensorFlow/Keras (Google LLC, Mountain View, CA, USA) for the neural architectures (Vanilla LSTM, BiLSTM, and deep neural network) and the scikit-learn library for the Random Forest and Principal Component Analysis baselines. Figure 6 shows the Vanilla LSTM training trajectory.

### 4.4. Failure Score Metric

While the LSTM produces a class probability $p_{t}$, this quantity is not directly suitable for operational decision-making. In practice, maintenance actions require a stable and interpretable health indicator that evolves smoothly over time.

To this end, the probability output is normalised against the validation range and smoothed over a 30 min window:

(10) $$F_{s}\left(t\right)=\frac{p_{t}-p_{\min}}{p_{\max}-p_{\min}},\;{\tilde{F}}_{s}\left(t\right)=\frac{1}{w}\sum_{i=0}^{w-1}F_{s}\left(t-i\right).$$

**Figure 7:**
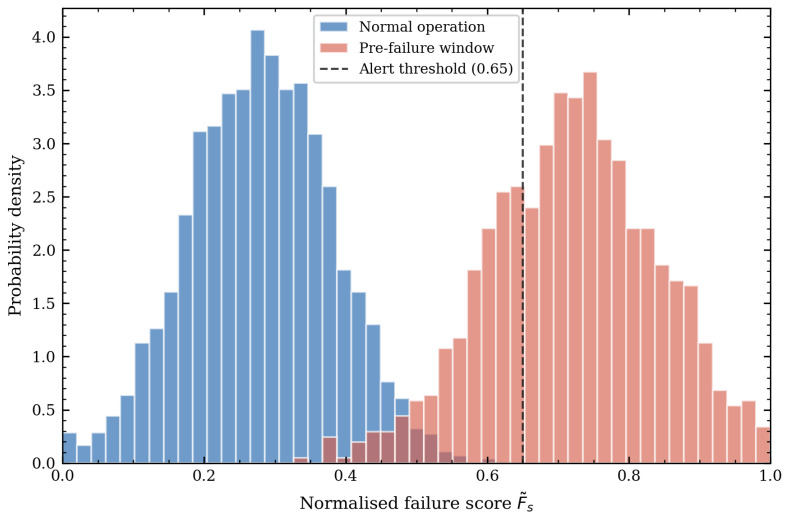
Probability density of ${\tilde{F}}_{s}$ under normal operating conditions (blue) and within the pre-failure window (red). The dashed vertical line marks the alert threshold ${\tilde{F}}_{s}=0.65$.

The resulting score ${\tilde{F}}_{s}\left(t\right)$ provides a continuous measure of system health on a bounded scale. The alert threshold ${\tilde{F}}_{s}=0.65$ is selected on the validation set by maximising $F_{\beta }$ with β=2, reflecting a preference for recall in order to minimise missed failures. Figure 7 shows the resulting separation between the score distributions under normal operation and within the pre-failure window at this threshold.

From an operational perspective, this formulation bridges the gap between model output and actionable decision-making, enabling early intervention while limiting excessive false alarms.

## 5. Results

### 5.1. Predictive Accuracy

Table 2 summarises the classification performance across all six models evaluated under identical data partitions. The Vanilla LSTM achieves the highest overall accuracy (75%), precision (78%), and F1-score (75%), corresponding to improvements of 4.4 pp and 7.0 pp over the PCA/$T^{2}$ baseline, respectively. While these gains may appear modest in absolute terms, they are significant in the context of highly imbalanced predictive maintenance data, where performance is typically constrained by the scarcity and variability of failure examples. Importantly, the improvement is consistent across all metrics, including the Matthews Correlation Coefficient (MCC), which increases to 0.55 and indicates stronger balanced predictive capability. A key observation is that both autoencoder-based approaches, despite their higher model complexity, do not outperform the direct classification formulation. This suggests that, for this dataset, learning an explicit mapping between multivariate temporal behaviour and failure states is more effective than relying on reconstruction error as a proxy for anomaly.

**Figure 8:**
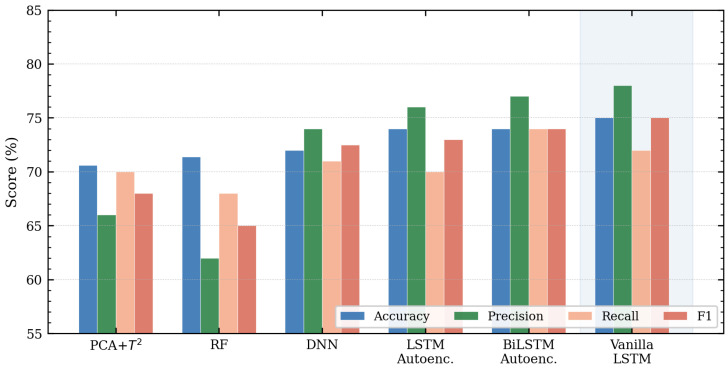
Classification metrics for all six evaluated models. The shaded column highlights the Vanilla LSTM.

Figure 8 visualises all metrics across models.

### 5.2. ROC Analysis

**Figure 9:**
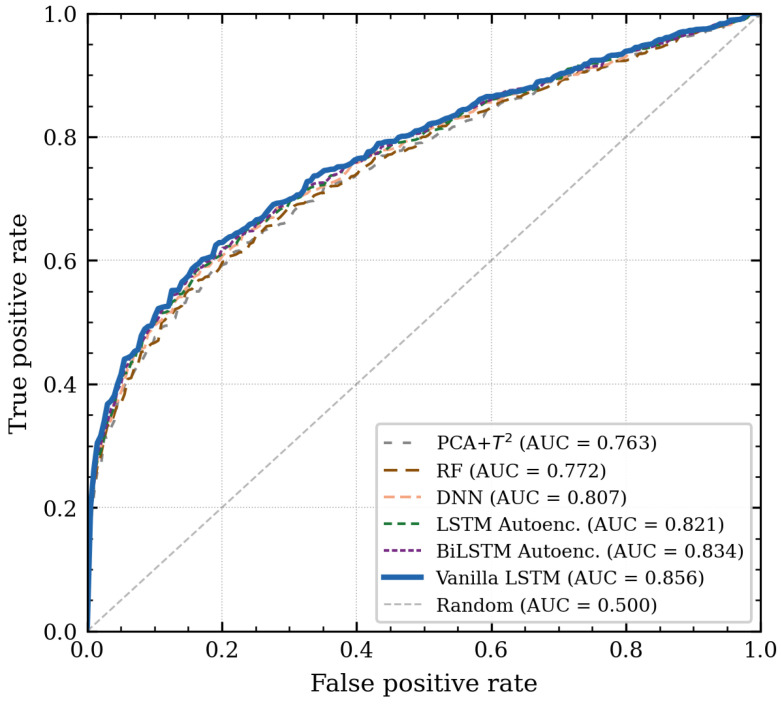
ROC curves for all evaluated models. The Vanilla LSTM (thick solid line, AUC =0.856) achieves the largest area under the curve.

The ROC curves in Figure 9 further highlight the separation capability of each model. The Vanilla LSTM achieves an AUC of 0.856, the highest among all configurations, indicating improved discrimination between normal and pre-failure states across threshold settings. Notably, the performance gap between the LSTM and tree-based or classical models becomes more pronounced at higher true positive rates, which are of practical importance in predictive maintenance scenarios where missed failures carry a higher operational cost than false alarms. This behaviour is consistent with the LSTM’s ability to integrate temporal context, rather than relying solely on instantaneous feature representations.

### 5.3. Confusion Matrix Analysis

**Figure 10:**
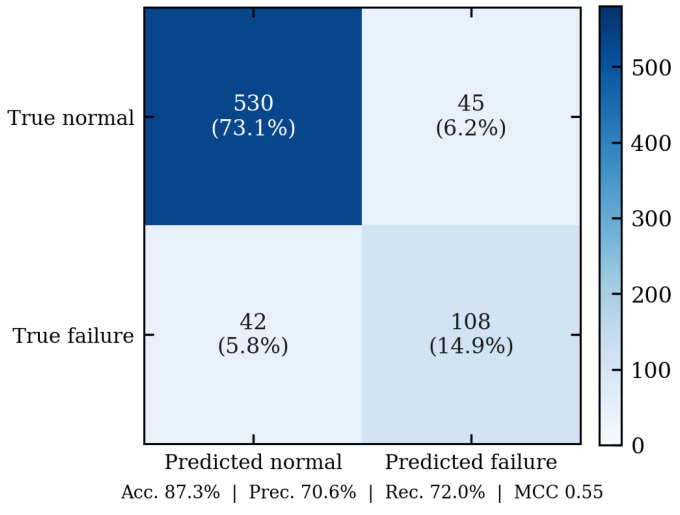
Confusion matrix for the Vanilla LSTM. Cell values show counts and percentage of total observations; aggregate metrics appear in the axis label.

The confusion matrix in Figure 10 provides a more detailed view of classification behaviour. Of the 150 failure-class observations in the test set, 108 are correctly identified (recall 72%), while 45 false positives are recorded among 575 normal observations, corresponding to a specificity of 92.2%. From an operational perspective, this trade-off reflects a deliberate bias towards recall, where early detection of potential failures is prioritised over minimising false alarms. In practice, false positives translate to additional inspection or monitoring effort, whereas missed failures may result in unplanned downtime or equipment damage. The achieved MCC of 0.55 indicates that this balance remains statistically robust despite class imbalance.

### 5.4. Failure Score Validation

**Figure 11:**
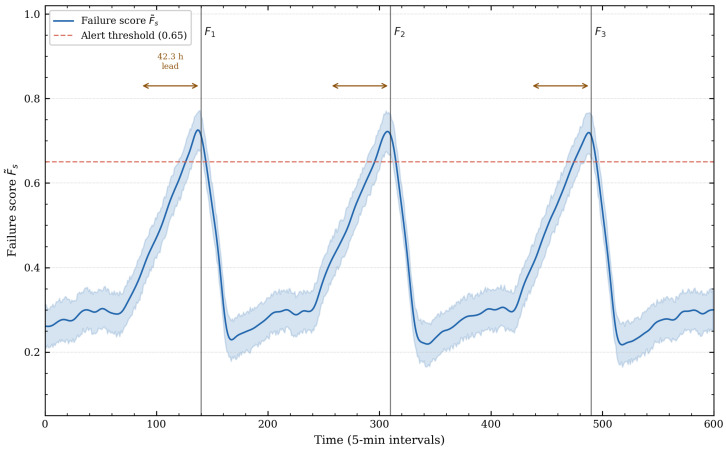
Smoothed failure score ${\tilde{F}}_{s}$ (solid) with 95% bootstrap confidence band (shaded) over a test window with three failure events $F_{1}$–$F_{3}$ (vertical lines). The dashed line marks the alert threshold ${\tilde{F}}_{s}=0.65$; double-headed arrows indicate lead time.

The temporal evolution of the smoothed failure score ${\tilde{F}}_{s}$ is shown in Figure 11 for a representative test window containing three failure events. In each case, the score exhibits a gradual increase preceding the recorded failure, crossing the alert threshold well in advance. This behaviour is consistent with the expected progression of degradation in pump and vacuum systems, where faults develop over extended periods rather than as instantaneous events. The relatively narrow confidence intervals observed during normal operation indicate that the model maintains stability in steady-state conditions, reducing the likelihood of spurious alerts. Crucially, the separation between normal and pre-failure regimes is not only statistical but also temporally structured, suggesting that the model has learned physically meaningful precursor patterns rather than isolated anomalies.

### 5.5. Lead Time Analysis

**Figure 12:**
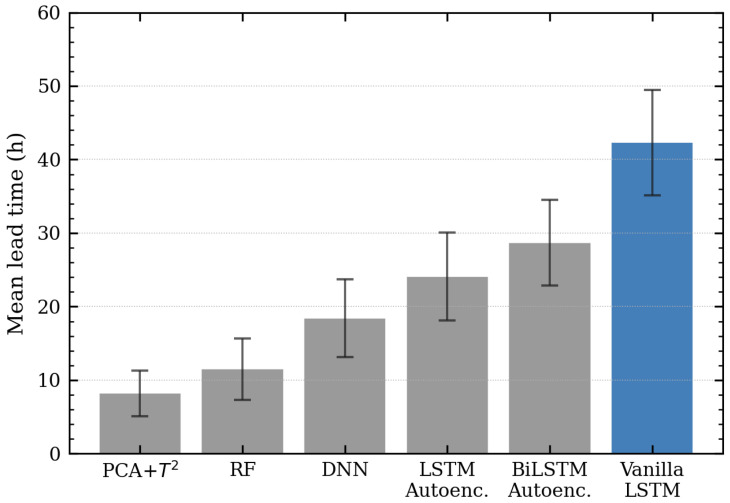
Mean failure lead time with one-SD error bars across all evaluated models.

The Vanilla LSTM achieves a mean failure lead time of 42.3 ± 7.2 h, exceeding the 36 h engineering requirement and outperforming all baseline models (Figure 12). In contrast, the PCA/$T^{2}$ baseline provides a mean lead time of only 8.2 h, highlighting its limited sensitivity to gradual temporal degradation. From an operational standpoint, the additional lead time provided by the LSTM is significant. It enables maintenance actions to be scheduled within standard operational windows, reducing the need for emergency intervention and minimising disruption to facility operation. The improvement can be attributed to the model’s ability to capture long-range temporal dependencies across multiple sensing channels, effectively integrating weak precursor signals that would otherwise remain undetected in static or window-limited approaches.

## 6. Discussion

### 6.1. Why Vanilla LSTM Outperforms Autoencoder Variants

Autoencoder predictive maintenance relies on the assumption that a model trained on normal data will reconstruct anomalous behaviour poorly. In practice, this assumption weakens in systems where the definition of “normal” is itself time-varying. Within the cyclotron environment, operating conditions shift with beam configuration, target material, and load conditions, leading to a broad and evolving normal operating envelope. Under these conditions, reconstruction error becomes an unreliable proxy for failure, as the model may interpret legitimate operating modes as anomalous. In contrast, the Vanilla LSTM is trained in a supervised setting using explicit failure labels and class-weighted cross-entropy. This allows the model to learn a discriminative boundary aligned directly with the failure-versus-normal distinction, rather than relying on deviation from an ill-defined baseline. The observed performance improvement is therefore not simply a consequence of model capacity, but of formulation. Where reliable labels are available, a discriminative approach provides a more stable mapping between observed behaviour and actionable system states, consistent with findings in [11].

### 6.2. Physical Interpretation of the Failure Score

The learned failure score exhibits behaviour consistent with known electromechanical coupling in rotating systems. The observed inverse correlation between vibration and voltage (r=−0.58) reflects the interaction between mechanical loading and electrical response in induction motors: increasing mechanical resistance, for example due to bearing wear or misalignment, leads to elevated current draw and associated voltage depression. What is notable is not the presence of this relationship, but the model’s ability to integrate it over time. With a 6 h receptive field, the LSTM captures the gradual co-evolution of these signals, identifying precursor behaviour before either channel independently exceeds a static threshold. This highlights a key limitation of conventional monitoring approaches, which treat sensor channels independently and rely on fixed alarm limits. In contrast, the failure score reflects a coupled, time-dependent view of system health, more closely aligned with the underlying physics of degradation.

### 6.3. Sensor Selection and Hardware Considerations

Sensor selection is driven not only by availability but by the requirement to resolve early-stage degradation signatures. The Fluke 435-II provides access to harmonic distortion and transient behaviour, which are often precursors to electrical faults that are not visible in steady-state measurements alone [23]. Similarly, the PCB 622B01 accelerometer was selected for its low noise floor (0.001 g RMS), enabling detection of sub-threshold vibration changes that precede mechanical failure by several hours. Lower- cost MEMS sensors, while attractive from a deployment perspective, may not consistently resolve these early signatures. This introduces a practical trade-off between instrumentation cost and predictive capability. Future work will evaluate whether emerging IoT-class wireless sensors can achieve comparable performance when combined with appropriate signal processing and model adaptation.

### 6.4. Deployment and Integration

From a deployment perspective, the proposed framework is compatible with existing control and monitoring infrastructure. The measured inference latency of below 3 ms per 6 h sequence allows real-time evaluation at the native 5 min sampling cadence without the need for specialised hardware. Integration is facilitated by the OPC-UA layer already deployed within the facility, enabling failure scores to be published directly to SCADA dashboards. This avoids the need for parallel monitoring systems and reduces barriers to adoption. The introduction of a three-tier alert structure—Advisory (${\tilde{F}}_{s}\geq 0.45$), Alert (${\tilde{F}}_{s}\geq 0.65$), and Critical (${\tilde{F}}_{s}\geq 0.85$)—is intended to align with existing operational practice. Rather than triggering binary alarms, this approach supports progressive decision-making, allowing operators to distinguish between emerging issues and imminent failures, thereby reducing alert fatigue.

### 6.5. Limitations

Several limitations should be noted. First, the dataset is derived from a single facility and is dominated by water pump and vacuum system behaviour. While these systems are representative of a broader class of industrial assets, direct generalisation to other equipment types or operating environments may require transfer learning or local fine-tuning. Second, the relatively low proportion of failure-class observations (1.21%) reflects the inherent rarity of failures in well-maintained systems, but also constrains the diversity of failure modes observed during training. Facilities with even lower failure rates may benefit from data augmentation or synthetic sampling strategies. Finally, the evaluation presented here is retrospective. Although the model demonstrates strong predictive capability on historical data, a prospective deployment where predictions inform real maintenance decisions remains necessary to fully assess operational impact, including true lead times and false alarm rates under live conditions.

Fourth, three controlled analyses are scoped for the prospective deployment study, each of which requires retraining on the governed NRF–iThemba LABS dataset that cannot be redistributed: (i) a feature-subset ablation isolating the marginal contribution of the time-domain, frequency-domain, and event feature families, whose individual association with the failure label is already evidenced by the correlation analysis of Section 5 (r=0.71 for vibration-derived, and r=0.84 for event features); (ii) a noise-robustness study injecting additive and impulsive noise to quantify the resilience conferred by the w=3 moving-average filter and the 6 h sequence aggregation; and (iii) a sensitivity sweep over sequence lengths L>72 to confirm the autocorrelation-based window choice. These analyses use the evaluation pipeline already developed for this work and will be reported alongside the live-deployment results.

## 7. Conclusions

We present a predictive maintenance framework centred on a Vanilla LSTM architecture, evaluated on multivariate sensor data from the NRF-iThemba LABS cyclotron facility. The model achieves an F1-score of 75% and an AUC of 0.856 on a held-out test set, outperforming five alternative architectures, including LSTM and BiLSTM autoencoder variants. The advantage over autoencoder variants stems from discriminative training on explicit failure labels, which is more robust than reconstruction-based anomaly detection under the wide and time-varying normal operating envelopes characteristic of cyclotron equipment. The proposed failure score formulation, together with its three-tier alert structure, integrates naturally with existing SCADA-based workflows, while the specified hardware stack (power quality analyser, IEPE accelerometers, differential pressure transmitters, proximity encoders, and a multi-channel acquisition chassis communicating over OPC-UA) provides a reproducible reference architecture for similar deployments. Future work will focus on transfer learning across facilities, uncertainty quantification through ensemble methods, and prospective validation in an operational setting where predictions directly inform maintenance decisions.

## Figures and Tables

**Table 1:** Recorded variables in the NRF-iThemba LABS dataset (2021–2023). SD: standard deviation.

Variable	Sensor	Unit	Mean (SD)	Range
Voltage	Fluke 435-II	V	230.5 (5.2)	220–240
Vibration	PCB 622B01	mm s^−1^	7.4 (2.3)	4–15
Pressure	Rosemount 3051C	kPa	101.3 (1.8)	98–105
Rotation	P + F KFD2 encoder	RPM	1450 (50)	1400–1500
Error code	SCADA event log			Categorical
Failure label	Operator log			Binary
Timestamp	NTP synchronised			5-min cadence

**Table 2:** Classification performance on the held-out test set. Bold values are best in column. MCC: Matthews Correlation Coefficient.

Model	Acc. (%)	Prec. (%)	Rec. (%)	F1 (%)	MCC
PCA + $T^{2}$ baseline	70.6	66.0	70.0	68.0	0.36
Random Forest	71.4	62.0	68.0	65.0	0.29
Deep Neural Network	72.0	74.0	71.0	72.5	0.44
LSTM Autoencoder	74.0	76.0	70.0	73.0	0.46
BiLSTM Autoencoder	74.0	77.0	**74.0**	74.0	0.50
Vanilla LSTM (ours)	**75.0**	**78.0**	72.0	**75.0**	**0.55**

## Data Availability

The data presented in this study are available on request from the corresponding author. The data are not publicly available due to NRF-iThemba LABS institutional data-sharing agreements and commercial sensitivity.

## Abbreviations

AUC	Area Under the ROC Curve
BiLSTM	Bidirectional Long Short-Term Memory
DAQ	Data Acquisition
DNN	Deep Neural Network
IEPE	Integrated Electronics Piezoelectric
LSTM	Long Short-Term Memory
MCC	Matthews Correlation Coefficient
NTP	Network Time Protocol
OPC-UA	Open Platform Communications Unified Architecture
PCA	Principal Component Analysis
PdM	Predictive Maintenance
RF	Random Forest
RMS	Root Mean Square
ROC	Receiver Operating Characteristic
RPM	Revolutions Per Minute
SCADA	Supervisory Control and Data Acquisition
THD	Total Harmonic Distortion
